# Leptin and G-protein coupled receptor (GPCR) signaling: Therapeutic potential in obesity

**DOI:** 10.1016/j.jbc.2025.110768

**Published:** 2025-09-26

**Authors:** Xun Sun, Lincoln Brueck, Dongming Yang, Patrick L. Sheets, Baohua Zhou, Hongxia Ren

**Affiliations:** 1 Herman B. Wells Center for Pediatric Research, Indiana University School of Medicine, Indianapolis, Indiana, USA; 2 Center for Diabetes and Metabolic Diseases, Indiana University School of Medicine, Indianapolis, Indiana, USA; 3 Stark Neurosciences Research Institute, Indiana University School of Medicine, Indianapolis, Indiana, USA; 4 Department of Biochemistry, Molecular Biology, and Pharmacology, Indiana University School of Medicine, Indianapolis, Indiana, USA; 5 Department of Anatomy, Cell Biology and Physiology, Indiana University School of Medicine, Indianapolis, Indiana, USA

**Keywords:** GPCR, leptin, obesity, diabetes, signaling, energy balance, pharmacology

## Abstract

Leptin, an adipocyte-derived hormone, plays a crucial role in regulating food intake and energy homeostasis. However, individuals with obesity exhibit hyperleptinemia and impaired leptin responsiveness, which contribute to greater food intake, reduced energy expenditure, and metabolic dysregulation, exacerbating weight gain and obesity-related complications. Leptin resistance remains a major challenge in obesity treatment, limiting the efficacy of leptin-based therapies. G-protein coupled receptors (GPCRs) are a large family of seven-transmembrane (7TM) receptors that respond to a variety of ligands, including neuropeptides, gastrointestinal hormones, and metabolites. GPCRs are central regulators of glucose metabolism and energy balance, which have emerged as key drug targets for diabetes and obesity. Combining leptin with GPCR-targeting therapies, such as gut peptides, shows promise in overcoming leptin resistance and improving metabolic outcomes. Understanding the molecular crosstalk between leptin and GPCRs provides valuable insights for expanding leptin’s therapeutic potential and developing effective anti-obesity treatments. In this review, we highlight the therapeutic potential of combining molecules targeting GPCR signaling with leptin for obesity treatment.

## Leptin and obesity

### Leptin signaling pathway

Leptin is a hormone derived from adipose tissue and is required for energy homeostasis and body weight regulation (1, 2). As it was discovered 30 years ago, our understanding of leptin and its role in the pathophysiology of obesity has advanced significantly (3, 4, 5). Leptin levels positively correlate with body fat percentage and exhibit significant diurnal fluctuations (6, 7, 8). Leptin regulates glucose and lipid metabolism, exercise, immune function, and reproduction, and plays an important role in obesity and related metabolic diseases (9).

**Figure 1:**
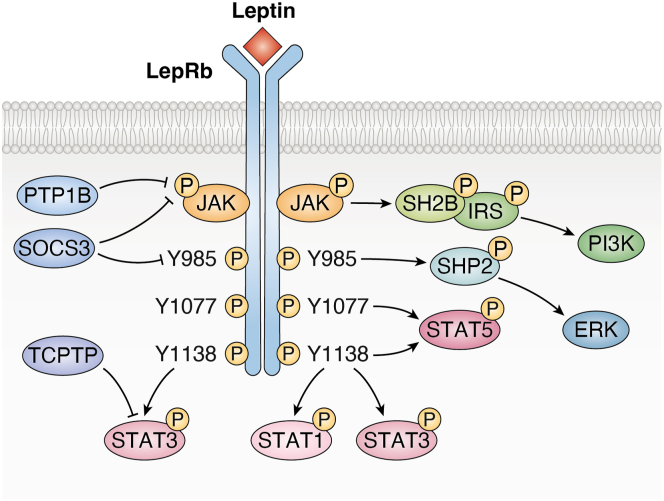
**Leptin signaling.** See text for explanatory details. LepRb, leptin receptor; JAK2, janus kinase 2; STAT1, signal transducer and activator of transcription 1; STAT3, signal transducer and activator of transcription 3; STAT5, signal transducer and activator of transcription 5; ERK, extracellular signal-regulated kinase; IRS, insulin receptor substrate; PI3K, phosphatidylinositol 3-kinase; SOCS3, suppressor of cytokine signaling 3; PTP1B, protein tyrosine phosphatase 1B; TCPTP, T-cell protein tyrosine phosphatase. This figure is created using BioRender.

Leptin exerts its biological effects primarily through the leptin receptor (LepR), which exists in multiple isoforms, with the long isoform (LepRb) being the critical mediator of leptin's physiological functions (10, 11). The key signaling pathway activated by leptin is the janus kinase 2 (JAK2)/signal transducer and activator of transcription 3 (STAT3) pathway (12). Upon leptin binding to LepRb, JAK2 is activated and phosphorylates STAT3 (13, 14). Additionally, tyrosine-phosphorylated LepRb can activate other signaling molecules, including STAT5, extracellular signal-regulated kinase (ERK), and insulin receptor substrate (IRS) proteins (15). The IRS proteins, in turn, activate the IRS/phosphatidylinositol 3-kinase (PI3K) pathway, which is also involved in insulin signaling, highlighting the overlap between leptin and insulin regulation of metabolic processes. Negative feedback regulation of leptin signaling is mediated by proteins such as suppressor of cytokine signaling 3 (SOCS3), protein tyrosine phosphatase 1B (PTP1B), and T-cell protein tyrosine phosphatase (TCPTP), which dephosphorylate the receptor and its downstream components (15, 16) (Fig. 1).

**Figure 2:**
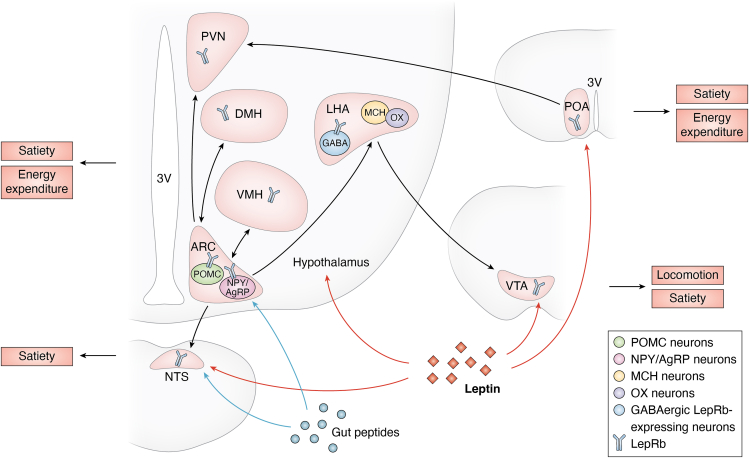
**The integrative regulation of hypothalamic circuits by leptin.** As described in the text, leptin acts at a number of regions in hypothalamus to regulate satiety, energy expenditure, and locomotion. The ARC contains anorexigenic POMC neurons and orexigenic NPY/AgRP neurons, which can both be acted on by leptin. There is a broad hypothalamic network in hypothalamus to coordinate responses to leptin’s action in food intake and energy status, which includes ARC, DMH, VMH, PVN, LHA, and POA. All regions show prominent LepRb expression. Outside the hypothalamus, LepRb is also expressed in the VTA and the NTS, and both VTA and NTS receive input from the hypothalamus. Except for GABAergic LepRb-expressing neurons, VTA contains orexigenic MCH and OX neurons. Gut hormones, as a group of key regulators on satiety, target NTS and ARC. ARC, arcuate nucleus; POMC; NPY/AgRP; DMH, dorsomedial hypothalamus; VMH, ventromedial hypothalamus; PVN, paraventricular nucleus; LHA, lateral hypothalamic area; POA, preoptic area; VTA, ventral tegmental area; NTS, nucleus of the solitary tract; GABA, gamma-aminobutyric acid; MCH, melanin-concentrating hormone; OX, orexins; 3V, third ventricle. This figure is created using BioRender.

LepRb is widely expressed in the central nervous system (CNS) and peripheral tissues, whereas leptin exerts long-term regulation on feeding behavior and energy metabolism primarily through the CNS, particularly in the hypothalamus (3, 17, 18). The mediobasal hypothalamus, particularly the arcuate nucleus (ARC) of the hypothalamus, is a critical site of leptin’s action with the highest density of LepRb, where it targets both anorexigenic pro-opiomelanocortin (POMC) neurons and orexigenic neuropeptide Y (NPY)/agouti-related protein (AgRP) neurons (19). These neurons are part of a broader hypothalamic network that communicates with other regions to coordinate responses to food intake and energy status, including the dorsomedial hypothalamus (DMH), ventromedial hypothalamus (VMH), paraventricular nucleus (PVN), lateral hypothalamic area (LHA), and preoptic area (POA), which also show prominent LepRb expression (20, 21, 22, 23). Outside the hypothalamus, LepRb is expressed in the ventral tegmental area (VTA), a structure controlling locomotion and appetite located within the midbrain. VTA can also receive input from LHA GABAergic LepRb neurons to suppress feeding (21, 24). Moreover, LepRb is also expressed in the nucleus of the solitary tract (NTS), which plays a crucial role in integrating signals conveyed by gut peptides and can be innervated by hypothalamus as well (22) (Fig. 2).

### Leptin and tissue inflammation in obesity

Adipose tissue in obesity is characterized by increased production of inflammatory cytokines and infiltration by macrophages and T cells. CD4 T cells express high levels of the long isoform of the leptin receptor LepRb, and leptin directly impacts the functional differentiation of CD4 T cells. *In vitro* leptin treatment promotes IL-2 and IFNγ production but reduces IL-4 production by CD4 T cells (25, 26), while LepR-deficient CD4 T cells produced less IFNγ than LepR-sufficient CD4 T cells (26). Leptin promoted Th17 differentiation of both human and mouse CD4 T cells by inducing RORγt transcription (27).

Regulatory T cells (Tregs) play a central role in maintaining immune system homeostasis and modulating immune responses. Tregs improve the inflammatory state and regulate metabolic homeostasis in adipose tissue and the hypothalamus. Compared to lean individuals, Treg cells were significantly reduced in the adipose tissue of obese patients and mice (28). Leptin inhibits Treg proliferation *in vitro,* and leptin neutralization antibody leads to increased Treg cell proliferation (29). Recently, a unique population of LepR + sympathetic perineurial barrier cells (SPCs) that ensheath adipose tissue sympathetic axon bundles have been identified in both humans and mice (30). The LepR + SPCs express IL-33 to modulate immune homeostasis in brown adipose tissue (BAT). Mice with SPC-specific deletion of IL-33 have less Tregs and eosinophils in BAT, resulting in increased BAT inflammation and being more susceptible to diet-induced obesity independent of food intake. Furthermore, mice with SPC-specific deletion of IL-33 have impaired adaptive thermogenesis and were unresponsive to leptin-induced rescue of metabolic adaptation (30).

The reciprocal interaction between the brain and the immune system affects the organismal metabolism (31). High-fat diet causes hypothalamic inflammation, which contributes to disrupted metabolic homeostasis and obesity (32). Mechanistically, high-fat, high-sugar diet reduces hypothalamic Tregs while activating hypothalamic CD4 T cells and infiltrating macrophages and microglia (33). Transient Treg depletion significantly enhances diet-induced hypothalamic inflammation, whereas *in vivo* Treg expansion by anti-IL2/IL2 antibody complex results in reduced body weight and fat mass gain, and improved glucose tolerance following high-fat and high-sugar diet (33).

### Leptin in obesity treatment

Although leptin is a key regulator of food intake and energy homeostasis, its therapeutic efficacy in obesity is limited in part by leptin resistance—a condition characterized by elevated leptin levels and reduced leptin sensitivity during times of nutritional abundance. This resistance diminishes leptin's ability to regulate appetite and energy expenditure, representing a major obstacle to the development of effective leptin-based obesity treatments. The underlying mechanisms of leptin resistance are multifactorial and may include impaired transport of leptin across the blood-brain barrier, altered leptin receptor function, and dysregulation of downstream signaling pathways in the hypothalamus and peripheral tissues (34).

Hormonal excess often results in resistance. Reducing leptin in circulation was proposed to curb leptin resistance. An earlier study showed that leptin heterozygosity reduced leptin levels in melanocortin receptor heterozygous mouse models and had beneficial effects on energy homeostasis (35). A partial reduction in plasma leptin levels by genetically eliminating leptin, neutralizing anti-leptin antibodies, or by weight loss compounds has been proposed as a potential strategy for promoting body weight reduction (36, 37, 38). On the other hand, sufficient leptin is also key to setting and maintaining energy homeostasis. During fasting or weight loss maintenance, an energy deficit leads to reduced leptin levels, which in turn trigger increased appetite, suppressed reproductive and thyroid hormone activity, and decreased energy expenditure (39, 40). The decline in leptin has been recognized as a physiologically critical signal of starvation (41). This mechanism helps explain the high relapse rate—up to 80%—among individuals who initially succeed in losing weight. Studies involving participants who achieved a 10% reduction in body weight through dietary interventions suggest that leptin insufficiency contributes to these adverse physiological responses. Restoring leptin levels to pre-weight-loss concentrations reversed these changes and supported sustained weight loss (42, 43, 44). Notably, animals with mutation or dysregulation in the leptin gene can lead to hypoleptinemic obesity, and this form of obesity is responsive to leptin treatment (45, 46).

For most obese patients, overcoming leptin resistance remains a primary objective in achieving weight loss by using leptin. Meanwhile, there is growing interest in developing leptin-based therapeutic strategies aimed at restoring leptin’s efficacy as a treatment for obesity. Notably, G-protein coupled receptors (GPCRs) have emerged as critical modulators in leptin sensitivity, offering new insights into the mechanisms of leptin resistance and potential therapeutic targets. In the next section, we will explore the interaction between GPCR signaling and leptin sensitivity and highlight the therapeutic potential of combining leptin and GPCR ligands, many of which are gut peptides, neurotransmitters, and neural peptides, for obesity treatment.

## GPCRs/GPCR ligands and leptin sensitivity

**Figure 3:**
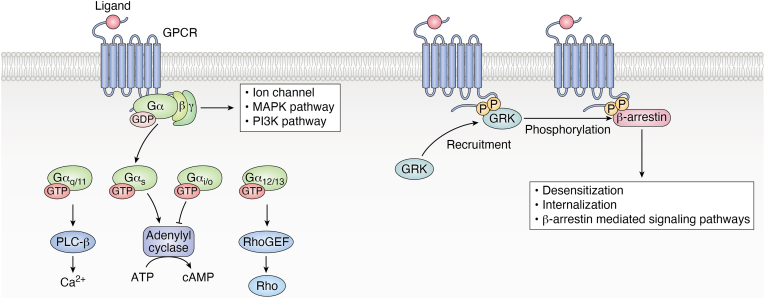
**GPCR signaling.** GPCRs are a large family of seven-transmembrane receptors. After binding with agonist ligands, GPCR couples to heterotrimeric G proteins that are composed of Gα, Gβ, and Gγ subunits. Gα subunit is further classified into four main families: Gα_q/11_, Gα_s_, Gα_i/o_, Gα_12/13_. The activation of GPCR leads to the production of second messengers and downstream effectors like Ca^2+^, cAMP, and Rho, triggering cellular responses. Additionally, activated receptors are phosphorylated by GRK, allowing them to bind to β-arrestins, promoting desensitization, internalization, and β-arrestin-mediated signaling pathways. GPCRs, G-protein coupled receptors; GRK, GPCR kinases; GDP, Guanosine diphosphate; GTP, Guanosine triphosphate; ATP, adenosine triphosphate; cAMP, cyclic adenosine monophosphate; Rho, Ras homologous, RhoGEF, Rho guanine nucleotide exchange factor. This figure is created using BioRender.

GPCRs represent the largest family of membrane receptors, playing pivotal roles in transducing extracellular signals into intracellular responses. These receptors are activated by a diverse array of ligands, including hormones, neurotransmitters, peptides, lipids, and even environmental stimulants (47). GPCRs are classified into six types (Class A-F) based on sequence and function, which is designed to cover all GPCRs in both vertebrates and invertebrates (48). The binding of an agonist ligand to a GPCR couples the GPCR to heterotrimeric G proteins that are composed of Gα, Gβ, and Gγ subunits. Gα subunit is further classified into four main families: Gα_s_, Gα_i/o_, Gα_q/11_, Gα_12/13_. The activation of GPCRs leads to the production of second messengers and downstream effectors, triggering cellular responses. Additionally, activated receptors are phosphorylated by GPCR kinases (GRK), allowing them to bind to β-arrestins, promoting GPCR internalization and desensitization, as well as β-arrestin-mediated signaling pathways (49) (Fig. 3). The structural diversity of GPCR ligands enables selective activation of specific receptor subtypes, leading to finely tuned physiological outcomes (50). Owing to their versatility and pharmacological accessibility, GPCRs are the targets of a large proportion of clinically approved drugs (51). Advances in ligand engineering and biased agonism have further expanded the therapeutic potential of GPCR-targeting compounds, paving the way for more precise and effective interventions (52, 53).

The activation of GPCRs plays a critical role in numerous metabolic pathways, including nutrient sensing, transport, storage, and insulin sensitivity. Consequently, GPCRs are closely associated with the development of major metabolic disorders such as metabolic syndrome, diabetes, dyslipidemia, and nonalcoholic steatohepatitis (54). A large family of metabolite GPCRs, which are expressed in enteroendocrine and pancreatic endocrine cells, metabolic organs, and immune cells, specifically recognizes and binds to metabolites and mediates their signaling (55). Beyond that, many receptors for gut peptides, pancreatic peptides, neuropeptides, and neurotransmitters are GPCRs. In the context of metabolic regulation and obesity, both endogenous and synthetic GPCR ligands have gained increasing attention for their ability to modulate signaling pathways involved in appetite control, energy expenditure, and hormone sensitivity (56, 57). Some of these GPCRs have been investigated as potential targets for obesity treatment and demonstrate physiological interaction with leptin signaling. We summarized recent studies on the physiological interactions between GPCR and leptin signaling, offering insights into the role of GPCR signaling in enhancing leptin responsiveness, primarily focusing on GPCR- and leptin-based therapeutic strategies for the treatment of obesity (Table 1).

**Table 1:** GPCRs are reported to influence leptin effects/sensitivity

GPCRs are reported to influence leptin effects/sensitivity	Endogenous ligand	Agonist or antagonist desired for obesity treatment	Synergizing with leptin
Amylin receptors (AMYRs) (61, 64)	Amylin	Agonist	Yes (64)
Cholecystokinin 1 receptor (CCK1R) (75, 76, 79, 81, 82, 83, 84)	Cholecystokinin (CCK)	Agonist	Yes (81, 82, 83, 84)
Glucagon-like peptide 1 receptor (GLP-1R) (92, 95, 96, 97, 101)	Glucagon-Like Peptide 1 (GLP-1)	Agonist	Yes (92, 95, 96, 97, 101)
Glucagon receptor (GCGR) (96)	Glucagon (GCG)	Agonist	Yes (96)
Glucose-dependent insulinotropic peptide receptor (GIPR) (101)	Glucose-dependent insulinotropic peptide (GIP)	Agonist/antagonist	Yes (101)
Neuropeptide Y-Y2 receptor (NPY-Y2R) (105, 106)	Peptide YY3-36 (PYY3-36)	Agonist	Yes (105, 106)
Growth hormone secretagogue receptor (GHSR) (110, 111)	Ghrelin	Antagonist	Unclear
Dopamine 2 receptor (D2R) (120)	Dopamine	Antagonist	Unclear
Serotonin receptors (5-HTRs), except for 5-HT3R (124)	Serotonin (known as 5-hydroxytryptamine, 5-HT)	Agonist	Yes (124)
Melanocortin 4 receptor (MC4R) (136)	α-melanocyte-stimulating hormone (α-MSH)	Agonist	Unclear
Neurotensin receptor 1, 2 (NTR1, NTR2) (149, 150, 151)	Neurotensin (NT)	Agonist	Yes (149)
Oxytocin receptor (OTR) (159, 160)	Oxytocin (Oxt)	Agonist	Unclear
Thyrotropin-releasing hormone receptor (TRHR) (170)	Thyrotropin-releasing hormone (TRH)	Agonist	Yes (170)
Cannabinoid 1 receptor (CB_1_R) (176, 177)	Cannabinoids	Antagonist	Yes (176)
Adenosine A2A receptor (A2AAR) (183)	Inosine	Agonist	Unclear
Takeda G-protein coupled receptor 5 (TGR5) (184)	Deoxycholic acid (DCA)	Agonist	Unclear
G-protein coupled receptor 17 (GPR17) (186, 188, 192)	Unclear	Antagonist	Unclear

### Gut peptides

#### Amylin

Amylin, which was discovered as the main constituent of pancreatic islet amyloid in 1986, belongs to the calcitonin peptide family and is mainly secreted from pancreatic beta-cell (58, 59). Amylin has been shown to reduce food intake, control energy homeostasis, and affect the development of certain brain areas that are involved in metabolic control. Amylin receptors 1 to 3 (AMY1-3) are formed by the interaction of the calcitonin receptor (CTR) with receptor activity-modifying proteins (RAMPs) 1, 2, or 3, respectively. CTR is a class B GPCR that binds the peptide hormone calcitonin, while RAMPs are single-pass transmembrane proteins. Recent reviews discuss amylin signaling and its physiological effects in detail (58, 60, 61).

The interaction between amylin and leptin signaling involves multiple brain regions, including the AP, NTS, LHA, VMH, ARC, and VTA, where amylin receptors are widespread (61, 62, 63). Amylin functions as an endogenous leptin sensitizer and acts synergistically with leptin to enhance the suppression of food intake and the reduction of body weight, as demonstrated in both preclinical and clinical studies (61). More specifically, amylin treatment enhances leptin signaling in the hypothalamus in both leptin-sensitive and leptin-resistant conditions (64, 65). A landmark preclinical and clinical study on the co-administration of pramlintide, the only currently FDA-approved amylin analog, and metreleptin, a recombinant human leptin analog, established the therapeutic strategy of leveraging natural synergies to promote greater weight loss (64). Mechanistically, this study showed that enhanced leptin signaling in the VMH and AP, along with reduced food intake, contributed to the observed synergistic weight loss effects. Subsequent rodent experiment identified VMH microglial IL-6 production induced by amylin as a likely mechanism by which amylin enhances leptin signaling and amplifies its weight-reducing effects (62). Additional findings indicate that both amylin and leptin activate the same population of AP neurons through cyclic adenosine monophosphate (cAMP) signaling, providing further insight into the molecular basis of their synergistic interaction (66). Moreover, the weight loss achieved through combined amylin and leptin treatment requires ongoing administration of both agonists and is associated with sustained improvements in body composition, lipid metabolism, and insulin sensitivity (67). However, development of pramlintide/metreleptin combination therapy was halted in 2011 after a randomized clinical trial was discontinued due to safety concerns.

Recent research into novel amylin analogs—such as cagrilintide, petrelintide, amycretin, and GUB014295—has focused on developing new treatments for diabetes and obesity, particularly in combination with GLP-1 receptor agonists. For instance, the coadministration of cagrilintide and semaglutide (referred to as CagriSema) has demonstrated significant and clinically meaningful reductions in body weight among obese adults, both with and without type 2 diabetes (68, 69). In addition, studies in rats suggest that approximately one-third of CagriSema’s weight-loss efficacy is attributable to increased energy expenditure and attenuation of metabolic adaptation, indicating a potential enhancement of leptin sensitivity (70). However, it remains unclear whether coadministration with leptin could further amplify the weight-loss effects of these emerging compounds.

#### CCK

Cholecystokinin (CCK) is a gut peptide secreted by enteroendocrine I cells in the proximal small intestine in response to meal ingestion and promotes satiety by reducing food intake and slowing gastric emptying (71). Due to its structural similarity to gastrin, CCK shares certain biological and pharmacological effects by interacting with cholecystokinin receptors (CCKRs), CCK1R and CCK2R, which are class A GPCRs (72). The CCK1 receptor contains both high- and low-affinity binding sites, which are responsive to different concentrations of CCK—picomolar (pM) levels for high-affinity sites and nanomolar (nM) levels for low-affinity sites (73). The anorexigenic effect of exogenous CCK is reported to be primarily mediated through activation of the low-affinity sites on the CCK1 receptor (74).

Studies have shown that activation of the CCK1 receptor through administration of the CCK agonist CCK-8 increases leptin concentrations in the cerebrospinal fluid (CSF), facilitates leptin access to hypothalamic regions, and thereby enhances leptin's action on hypothalamic targets involved in body weight regulation (75, 76). Notably, most nodose neurons that are sensitive to CCK also respond to leptin stimulation. It is generally accepted that the convergence of leptin and CCK signaling, mediated through ionic mechanisms, occurs within vagal afferent neurons (77). Furthermore, the majority of NTS LepR neurons receive direct input from CCK-sensitive C-type vagal afferents and subsequently project to other CNS regions involved in the regulation of food intake (78). Additionally, a rat study indicated that leptin’s effects on anxiety and exploratory behavior may also be mediated *via* the CCK1 receptor (79). Overall, leptin and CCK influence food intake through interactions with multiple neural systems—including viscerosensory, motivational, affective, and motor pathways—at several levels of the neuroaxis (80).

The synergistic effects of leptin and CCK on satiety have been widely documented (81, 82, 83, 84). Co-administration of leptin and CCK, either systemically or in specific brain regions, has been shown to enhance c-Fos expression (82, 83). Mechanistically, this synergy is thought to involve reduced AMP-activated protein kinase (AMPK) phosphorylation, increased expression of Cocaine- and Amphetamine-Regulated Transcript (CART) and TRH, and recruitment of neural pathways from the hindbrain to the hypothalamus (81). The outcomes of this coordinated signaling include delayed gastric emptying and short-term suppression of food intake, thereby contributing to satiety through feedback regulation (85).

#### GLP-1

Glucagon-Like Peptide 1 (GLP-1) is derived from pre-proglucagon, released from intestinal endocrine L cells, and inhibits food intake by acting in both the periphery and CNS (86, 87). GLP-1 receptor (GLP-1R) is a GPCR that helps regulate blood glucose and food intake in islet beta cells and the CNS (88, 89). GLP-1R agonists, a typical class B GPCR ligand, lead to significant weight loss in the clinic and are FDA-approved obesity therapies. GLP-1R and LepR are coexpressed in certain neurons, particularly in the hypothalamus, and this coexpression plays a role in regulating food behavior and body weight. For example, a GABAergic LepRb-GLP-1R neuron population, located primarily in the DMH, regulates food intake and promotes body weight reduction through leptin and GLP-1R agonists (90). Although a larger proportion of heterogeneous POMC neurons in ARC expressed GLP-1R or LepR in the absence of the other, 10.2% of POMC neurons expressed mRNAs of both receptors (91).

The combination of leptin with GLP-1 agonist exendin-4 (Ex4) has shown promising synergistic effects in suppressing appetite in rats, while this effect is abolished at high leptin doses (92). Mechanistically, the interaction between LepR signaling and GLP-1R signaling in the hindbrain appears to act in at least an additive manner to regulate food intake and body weight (93). Rats pretreated with leptin also show stronger satiety and weight loss in response to GLP-1 or Ex4 than GLP-1 or Ex4 alone. In contrast, fasting attenuated the anorexic response to GLP-1 or Ex4 through a leptin-dependent mechanism, suggesting the physiological plasma leptin level is a potent regulator of GLP-1-mediated satiety (94). Another study reports that the treatment of leptin analogs together with Ex4 or fibroblast growth factor 21 (FGF21) restores leptin responsiveness in DIO mice after an initial body weight loss of 30%. However, these mice must be switched from high-fat diet (HFD) to chow at the beginning of the therapeutic intervention to avoid the powerful leptin resistance-inducing effect of HFD exposure (95). Subsequently, the same group reported that GLP-1/glucagon co-agonism can restore leptin sensitivity even in mice maintained on an HFD, following a ∼15% body weight reduction induced by PEGylated GLP-1/glucagon treatment (96). An additive reduction in food intake and body weight has also been observed with the co-administration of leptin and another GLP-1 agonist, liraglutide. Specifically, the reduction in food intake is attributed to decreased meal frequency instead of meal size (97). A recent study reports a newly developed GLP-1R/LepRb dual agonist and demonstrates its effect on satiety and weight control through LepRb/GLP-1R neurons (98).

Glucose-dependent insulinotropic peptide (GIP) is a gut-derived hormone secreted by K cells in the proximal intestine, playing a key role in stimulating insulin secretion and regulating body weight *via* binding to its G-protein-coupled receptor, GIPR. Interestingly, both GIPR agonists and antagonists have been shown to improve metabolic outcomes in models of diabetes and obesity (99, 100). We reported that dual GLP-1R and GIPR agonist tirzepatide (Tzp) enhances leptin signaling in hypothalamic neurons and results in more weight loss (101).

#### Peptide YY

Peptide YY (PYY) is produced by enteroendocrine L cells in the ileum and colon. The predominant circulating form, PYY3-36, is generated by DPP-4–mediated cleavage of full-length PYY. The anorectic effects of PYY3-36 are mediated through NPY-Y2R, a class A GPCR, with the hypothalamus, vagus nerve, and brainstem implicated as key target sites (102).

PYY shares functional similarities with leptin, as both reduce food intake by modulating appetite-regulating circuits in the hypothalamus. However, their actions regarding obesity differ significantly. In obese individuals, there is a marked resistance to leptin, whereas the anorectic effects of PYY are largely preserved. Moreover, endogenous PYY levels are reduced in obesity, suggesting that PYY deficiency may contribute to the pathogenesis of the condition (103). PYY levels rise postprandially, exerting short-term appetite-suppressing effects independently of leptin regulation (104). However, chronic leptin infusion in ad libitum–fed rats was shown to prolong the anorectic activity of PYY3-36 for up to 6 days, leading to a greater reduction in weight gain than either PYY3-36 or leptin alone (105). Co-treatment strategies combining long-acting PYY analogs with GLP-1 receptor agonists hold promise as effective anti-obesity therapies. One study demonstrated that the additive weight loss observed with PYY and Ex4 co-treatment in DIO mice was mediated by synergistic upregulation of appetite-regulating genes in the AP and improved leptin sensitivity (106).

#### Ghrelin

Ghrelin is an orexigenic peptide released in the stomach, with functions that regulate growth hormone release and control energy homeostasis by increasing food intake. The primary target for ghrelin to increase appetite is the growth hormone secretagogue receptor (GHSR), a class A GPCR, in the hypothalamus (107). The strategies targeting the ghrelin-GHSR system for obesity and metabolic diseases include neutralization of ghrelin, antagonism of ghrelin receptor by its antagonists and inverse agonists, inhibition enzyme involved in ghrelin binding to GHSR, as well as a potential pharmacological target to decrease ghrelin secretion (108).

Ghrelin and leptin act in parallel at the hypothalamic network to achieve an opposite outcome on feeding behavior (109). This is partly achieved by ghrelin increases SOCS3 expression and inhibits leptin-stimulated STAT3 phosphorylation in nodose ganglia (110). Reducing the constitutive activity of GHSR1a through inverse agonists has been reported to improve the sensitivity of anorectic leptin signals (111).

### Neurotransmitters and their cognate GPCRs

#### Amino acids

Glutamate and gamma-aminobutyric acid (GABA) are the two most common neurotransmitters in the CNS and play crucial roles in regulating neuronal excitability. Glutamate receptors and GABA receptors are widespread in the CNS, and the metabotropic subtypes (mGluRs and GABA_B_R) of these receptors are GPCRs. The ablation of the metabotropic glutamate receptor subtype mGlu5 in mice results in lower body weight and decreased plasma insulin and leptin concentrations under a high-fat diet (112). LepRb in GABAergic neurons mediates part of leptin's action in reducing hyperglycemia and body weight regulation (113, 114). Of note, a recent study reported that the activation of Arc GABA non-LepR neurons triggered massive obesity and leptin resistance with intact leptin-pSTAT3 signaling, suggesting a complex neural interaction between GABAergic neurons and LepRb (115). A pilot study showed that baclofen (GABA_B_R agonist) administration reduced body weight and serum leptin levels in obese patients with no significant changes in blood pressure or metabolism of glucose and lipid, which suggests targeting GABA_B_R might be a novel approach for obesity treatment (116). However, further studies are required to examine the interaction between GABA_B_Rs and leptin sensitivity.

#### Dopamine

The physiological roles of neurotransmitter dopamine (DA) in the brain are mediated by dopamine receptors, which are divided into two families based on G protein coupling: D1-like (D1 and D5) and D2-like (D2, D3, and D4), as D1-like receptors couple to stimulatory Gαs proteins and D2-like receptors couple to inhibitory Gαi proteins, respectively (117). Leptin has multiple distinct effects on the mesolimbic DA system. One evidence is that LepR is expressed in a minority of DA neurons in the VTA, a critical region of the mesolimbic DA system, and mediates leptin’s action on food intake and reward by decreasing the firing of VTA DA neurons (118). Besides that, leptin promotes the expression of tyrosine hydroxylase (TH) and the production of DA, which may be contributed by the innervation from LHA LepRb neurons (24).

Dopamine D2 receptors (D2Rs) are expressed in mouse and human adipocytes. D2R agonist quinpirole increases protein and mRNA expression of leptin *in vitro* and increases serum leptin concentration *in vivo* (119). D2R knock-out mice show reduced body weight and food intake with increased energy expenditure and decreased serum leptin level (120). Meanwhile, these mice are more sensitive to exogenous leptin, evidenced by increased hypothalamic pSTAT3 expression. Conversely, activating D2Rs by quinpirole suppressed the leptin-induced STAT3 phosphorylation in the hypothalamus of wild-type mice. This study also demonstrates the colocalization of D2Rs and pSTAT3 in the ARC. Leptin-induced food intake and body weight changes could be modulated by the treatment of D2R agonist and antagonist in wild-type mice but not in D2R knock-out mice. This study demonstrated that dopamine and leptin signaling pathways physiologically interact to regulate energy balance in the hypothalamus (120).

#### Serotonin

Central serotonin (also known as 5-hydroxytryptamine, 5-HT) signaling in mammalian species is involved in both behavioral and physiological determinants of energy homeostasis (121). All 5-HT receptors except for the 5-HT3 receptor are GPCRs. Serotonin interacts with leptin in regulating food intake, as leptin’s anorectic effect is partly mediated by projections from LepRs expressed serotonergic raphe neurons (DRN) to ARC (122). Sibutramine is a medication that was previously used to treat obesity by inhibiting the reuptake of serotonin and norepinephrine. Study shows the synergistic effect of sibutramine and leptin in decreased food intake and body weight reduction in diet-induced obesity (DIO) rats (123). Although sibutramine was withdrawn from the market in 2010 due to cardiovascular risks, it provides valuable insights into targeting the 5-HT system as a strategy to enhance leptin sensitivity. Hereafter, co-administration of leptin with meta-chlorophenylpiperazine (mCPP), an agonist of 5-HT2C and 5-HT1B receptors, has been shown to produce an additive effect in reducing body weight in DIO mice. Mechanistically, mCPP enhances leptin-induced STAT3 phosphorylation in the ARC, VMH, and ventral premammillary nucleus (PMV) (124).

### Neuropeptides

#### POMC

Neuropeptide receptors are part of the class A GPCR family. POMC is a large polypeptide precursor that gives rise to several biologically active peptides, including adrenocorticotropic hormone (ACTH), β-endorphin, and α-, β-, and γ-melanocyte-stimulating hormones (MSH) (125). α-MSH signals through GPCRs, melanocortin four receptor (MC4R) and melanocortin three receptor (MC3R). POMC neurons, primarily located in the ARC, are highly heterogeneous and play crucial roles in reducing food intake and increasing energy expenditure when in states of positive energy balance (126). Another neuronal population in ARC, AgRP neurons, exerts opposing effects on metabolic regulation by releasing the inhibitory neurotransmitter GABA, along with the orexigenic peptides NPY and AgRP to promote foraging behavior and food intake during states of energy deficit (127). Thus, these two neuronal populations coordinate the regulation of feeding and metabolism in response to the body's energy status (128). Additionally, both populations are involved in the melanocortin system, which plays a critical role in regulating energy homeostasis and feeding behavior, as POMC neurons release α-MSH, and AgRP neurons release AgRP (129), a natural antagonist for MC3R and MC4R.

Leptin directly or indirectly acts on ARC POMC neurons, with approximately 30% of these neurons expressing the LepR (91, 130). A recent review has outlined leptin’s physiological effects on POMC neurons in detail (131). Leptin increases the release of α-MSH, a key hormone in mediating leptin actions by binding to and activating MC4R in PVN (132). The leptin-melanocortin pathway is central to energy metabolism and body weight regulation and is defined as the primary relationship between leptin and POMC.

Due to the intricate link with leptin actions, the central melanocortin system has been recognized as a useful drug target to combat obesity and other leptin-resistant related disorders. POMC neuron-specific G-protein-signaling modulator 1 (GPSM1) deficiency mice are protected against diet-induced obesity with decreased food intake and increased brown adipose tissue thermogenesis. Mechanistically, GPSM1 ablation in POMC neurons improves leptin sensitivity by increasing POMC expression and α-MSH production through PI3K/AKT/mTOR signaling (133). One study reports that the deficient endogenous melanocortin activation by leptin may be one element of the central leptin resistance, and melanocortin agonist MTII evokes potent anorexic and energy-enhancing responses (134). Although multiple melanocortin receptor agonists have been investigated, setmelanotide is currently the only MC4R agonist approved by the FDA for the treatment of specific genetic forms of obesity, including deficiencies in POMC, PCSK1, and LepR, as well as Bardet-Biedl syndrome (BBS) (135, 136). Peripherally administered setmelanotide crosses the blood–brain barrier, binds to MC4R in PVN and LHA, and helps to restore the function of the MC4R pathway, resulting in appetite suppression, improved insulin resistance, and increased resting energy expenditure (137). While setmelanotide is currently approved only for rare genetic types of obesity, its ability to reduce leptin resistance suggests potential for use in non-genetic forms of obesity and in combination therapy with leptin, both of which warrant further investigation in future studies.

#### NPY

NPY is one of the most potent orexigenic and energy-regulating peptides in the brain. It is structurally similar to polypeptide YY (PYY) and pancreatic polypeptide (PP), which are primarily expressed in the intestine (138, 139). These peptides constitute the NPY peptide family and exert their effects through Y receptors (Y1, Y2, Y4, Y5, and Y6), a family of class A GPCRs, with varying affinities. The NPY system is implicated in the physiological dysregulation associated with several disorders, including alcoholism, obesity, and anorexia. A review article discusses the specific physiologic roles of Y receptors and highlights their therapeutic potential in the treatment of obesity (140).

NPY is a key downstream target of leptin action in the brain, as leptin administration inhibits both NPY expression and secretion (141). Although NPY-deficient mice exhibit normal food intake and body weight, they display increased sensitivity to leptin treatment with a greater reduction in food intake and body weight compared to controls, indicating that Leptin and NPY work in a homeostatic loop (142). Y1 receptor is a major mediator of NPY-induced hyperphagia (143). When the ventromedial nucleus is blocked, there is an increase of NPY-Y1 receptor expression in the hypothalamus and contributes to increased levels of leptin and leptin resistance, commonly seen in obese individuals (144). Notably, the transcription of Y1 and Y5 receptor genes is from opposite strands of the same DNA sequence (145). Both Y1 and Y5 receptor antagonists are being explored as a potential therapeutic strategy for obesity and related metabolic disorders by influencing satiety and energy balance, while the direct link between Y1/Y5 receptor antagonisms and leptin resistance is not fully elucidated (140). In contrast, the agonists of Y2 and Y4 receptors, high-affinity receptors for PYY and PP, respectively, are noted as a potential anti-obesity treatment. The interaction between PYY and leptin sensitivity is discussed in the gut peptides section.

#### Neurotensin

Neurotensin (NT) is a neuropeptide expressed in the CNS and periphery as a central feeding inhibitor and associated with maintaining energy homeostasis. Specific activity of NT expression is dependent upon where it is produced and which NT receptor subtype (NTR1, NTR2, and NTR3) it binds to (146). NTR1 and NTR2 are GPCRs for the peptide hormone NT, while NTSR3, also known as Sortilin1 (SORT1), is a different type of receptor with a single transmembrane domain that binds NT with high affinity.

Approximately 60% of the LepR-expressing neurons in the LHA express NT and are classified as GABAergic LepRb NT neurons. These neurons can project to the VTA and substantia nigra compacta and are important for leptin-mediated control of ingestion and locomotor behaviors (147, 148). A study investigated the effects of NT and leptin on food intake using intracerebroventricular (ICV) injections in normal Long-Evans rats. NT alone induced a short-lasting reduction in spontaneous food intake, with a significant effect observed at 30 min post-injection. In contrast, the anorexigenic effect of leptin emerged only after 24 h. When co-administered, leptin significantly enhanced the early effect of NT at 30 min and prolonged its inhibitory action for an additional 30 min. Additionally, NT amplified leptin’s effects at both 30 and 60 min, but not at 24 h (149). NTR1 deficiency in mice or NT receptor antagonist treatment attenuates exogenous leptin-induced robust reductions in food intake, indicating the lack or block of NTR reduces sensitivity to the anorectic action of leptin (150, 151). The overall findings from these studies indicate the crossover of the NT and leptin systems and their synergistic effect on anorectic behavior. However, the short half-life of NT *in vivo* has hindered its development as a therapeutic agent for obesity. Notably, a study suggests that the pegylated analog of the NT peptide (P-NT) and liraglutide synergize to reduce food intake and body weight in DIO mice through a melanocortin-dependent pathway with decreased leptin levels and improved tolerability compared with liraglutide monotherapy (152). Moreover, NT has been implicated as a potential regulator to combat the comorbid chronic pain-obesity epidemic, with its promoting weight loss (anorexigenic) and providing pain relief (analgesic) (153).

#### Oxytocin

Oxytocin (Oxt) is a hypothalamic nonapeptide that has been known for controlling maternal behavior, nervous system development, β-cell responsiveness, and lipid metabolism. Signaling of oxytocin is conveyed *via* stimulation of the oxytocin receptor (OTR), a GPCR (154). Beyond that, Oxt is now well-recognized as an anorexigenic neuropeptide that can reduce gastric emptying, GI transit, and food intake (155). As early as 1989, the anorexigenic effect of Oxt has been reported by intracerebroventricular (ICV) and peripheral Oxt treatment in rats (156). Multiple studies identified that Oxt activity in food regulation is mediated *via* pathways of Oxt signaling in the vagus nerve and hindbrain. More specifically, vagus afferent neurons project towards the NTS to increase c-Fos expression, which serves as a marker for activated neurons, leading to suppression of food intake (157, 158).

Oxt administration has been extensively investigated for the treatment of obesity in both animal models and humans, with studies also reporting a significant interaction with leptin in the regulation of food intake (159). Oxt treatment has also been shown to overcome leptin resistance by reducing body weight and fat mass in DIO animals, as well as in rodents with impaired leptin receptor signaling. In mice with obesity-induced leptin resistance, chronic Oxt treatment improved the short-term satiety effects of leptin, but this outcome eventually reversed, and oxytocin did not maintain sensitivity for a chronic period (160). Another study of oxytocin treatment in leptin-resistant db/db mice resulted in an amelioration of obesity *via* vagal afferent neurons not requiring leptin actions (158). Researchers propose that this circumvention of leptin resistance occurs because of peripheral Oxt activating the satiety-related regions of the hindbrain, the NTS and area postrema (AP) (161). Several review articles provide further insights into the translational and therapeutic potential of oxytocin as an anti-obesity intervention (162, 163, 164). Although intravenous or intramuscular Oxt has a well-established clinical history, its effectiveness as a long-term weight loss strategy remains to be determined.

#### Other neuropeptide and neurotransmitter

Orexins (orexin-A and orexin-B) are hypothalamic neuropeptides that play a critical role in the regulation of feeding behavior by acting on two GPCRs, HCRTR1 and HCRTR2 (also known as OX1R and OX2R) (165, 166). Orexin-producing neurons in the LHA have been shown to regulate corticosterone release and participate in the hypothalamic–pituitary–adrenal (HPA) axis, coordinating behavioral and physiological responses to stress. Interestingly, leptin has been shown to inhibit both orexin neuronal activity and orexin-mediated stress responses *via* a network of leptin-sensitive neurons in the LHA (167). In the DRN, HCRTR1 is highly expressed in glutamatergic Vglut3 neurons, which project to the LHA and integrate into the canonical feeding circuitry. These DRNVglut3 neurons have been identified as downstream targets of leptin signaling and represent a potential therapeutic target for obesity. Administration of the HCRTR1 antagonist CVN45502 resulted in reduced food intake and body weight in both leptin-resistant DIO mice and leptin-deficient ob/ob mice, supporting the therapeutic potential of modulating this pathway (168).

Thyrotropin-releasing hormone (TRH) regulates energy homeostasis not only *via* its effects on thyroid function but also through central actions on feeding, thermogenesis, locomotion, and autonomic control (169). Thyrotropin-releasing hormone receptor (TRHR) belongs to the class A GPCRs and plays a crucial role in the hypothalamic-pituitary-thyroid axis. Hypophysiotropic TRH neurons, located in the PVN, receive direct monosynaptic projections from leptin-responsive POMC and NPY/AgRP neurons in ARC (169). Beyond the hypothalamus, a cooperative interaction between leptin and TRH has been identified in the regulation of thermogenesis through hindbrain pathways. Specifically, this interaction enhances thermogenic responses in BAT (170). Notably, the synergy between leptin and TRH appears to be order-specific, as elevating leptin levels either through exogenous administration or refeeding before TRH injection leads to a significantly greater increase in BAT thermogenesis (171) Furthermore, leptin pretreatment significantly enhances the responsiveness of neurons in NTS to TRH, further supporting a functional synergy between these two hormones in central energy regulation (172).

Leptin and neurotransmitter endocannabinoids have opposite effects on appetite and energy balance. Two subtypes of cannabinoid receptors, type 1 (CB_1_) and type 2 (CB_2_), both belong to the GPCR family and play key roles in the CNS and immune system, respectively (173, 174). Cannabinoid receptor antagonists, particularly CB_1_ receptor (CB_1_R) antagonists, can lead to weight loss by suppressing appetite and affecting energy metabolism. Rimonabant is an early CB1R antagonist that showed promise for obesity treatment and was approved in Europe in 2006. However, it was withdrawn 2 years later due to psychiatric side effects (175). A rodent study showed that combining low doses of rimonabant and leptin significantly reduced body weight in DIO rats, with effects greater than those achieved by either agent alone. This enhanced effect is attributed to synergistic modulation of neuronal activity within the hypothalamus (176). Another study reported that the hypothalamic STAT3 signaling initiated by leptin can be diminished by agonists of cannabinoid receptor 1 (CB_1_R). *In vitro* experiment shows that CB_1_R activation engages β-arrestin1 to coordinate the TCPTP-mediated inhibition of hypothalamic STAT3 signaling, suggesting a direct target of the leptin signal transduction (177).

### Metabolites and their receptors

Various metabolites act through metabolite-sensing GPCRs to influence leptin secretion and signaling pathways. Although body fat is the primary driver of leptin synthesis, studies have demonstrated that short-chain fatty acids (SCFAs), ligands of GPR41 (also known as free fatty acid receptor 3, FFAR3), stimulate leptin expression in adipocytes (178, 179). However, a contradictory report indicates the role of Ga(i) signaling mediated by GPR43 (FFAR2), instead of GPR41, in SCFA-stimulated leptin secretion (180). In mice with lysophosphatidic acid receptor subtype (LPA1R) ablation, the serum leptin level and leptin mRNA level in adipocytes are significantly increased (181). These results demonstrated the important role of bioactive lipids in leptin secretion.

Inosine, a breakdown product of purine adenosine, acts as an agonist for the adenosine A2A receptor (A2AAR). It is serving as a critical regulator of immune checkpoint inhibition therapeutic response in various tumor types, and has been discovered to increase energy expenditure, browning of adipose tissue, and improve leptin sensitivity (182). A study reports that GLP-1RAs partially reversed obesity-induced hypothalamic leptin resistance through microbiota-driven inosine. Furthermore, inosine treatment reversed obesity-induced hypothalamic leptin resistance and ameliorated obesity through the leptin pathway (183).

A recent study examined the infusion of Takeda G-protein coupled receptor 5 (TGR5) agonist or ligands deoxycholic acid (DCA) in the NTS reverses high-fat diet-induced leptin resistance by enhancing leptin-STAT3 signaling to lower food intake (184).

### Other GPCRs

Evolving studies bring new insights into investigating orphan GPCRs in obesity therapeutic applications (185). Along with dedicating to identifying the natural ligands of numerous orphan GPCRs, extensive studies show the pathophysiology of orphan GPCRs in obesity and their interaction with leptin signaling.

Our group has identified that G-protein coupled receptor 17 (GPR17) is a transcription target of Forkhead box protein O1 (FoxO1) in the hypothalamus (186). The deletion of GPR17 in AgRP neurons increases satiety during fasting-refeeding challenge, and its ablation in POMC neurons increases aMSH processing and POMC neurons firing (187, 188). We also show that GPR17 is co-expressed in GLP-1-expressing enteroendocrine cells (EECs) in human and rodent intestinal epithelium and modulates nutrient-induced GLP-1 secretion (189). The *in vitro* experiment reveals that human GPR17 long isoform (hGPR17L) in GLUTag cells regulates GLP-1 secretion dependent upon Gα_i/o_ signaling instead of Gα_q_ signaling (190). Moreover, gut microbiota modulates the expression level of GPR17 and contributes to leptin resistance, suggesting the integration of microbiota in the brain-gut axis and the potential involvement of GPR17 (191). To investigate the metabolic function of GPR17 in leptin signaling, we generated GPR17 conditional knockout mice in leptin receptor-specific neurons and observed increased energy expenditure and oxygen consumption. GPR17 Knockout mice under a leptin-deficient background responded to exogenous leptin with reduced food intake and increased hypothalamic pSTAT3 activation, indicating improved leptin sensitivity (192).

Neuronal cilia, which play a key role in energy homeostasis, can be associated with obesity in the presence of ciliopathies, a subset of genetic disorders of the cilia. Most GPCRs exert their functions at cilia. Several of the GPCRs mentioned above, like MC4R, NPY2R, NPY5R, 5HT6, and DRD1, localize to cilia on neurons deep within the brain. In obese ciliopathy mouse models, the ciliary localization of these receptors is disrupted, suggesting a link between impaired ciliary signaling and obesity (193). Primary cilia for energy regulation, which are neuronal cilia that sense environmental cues and transduce extracellular signals within the cells, are often found in the hypothalamus. Trafficking of LepR is achieved by primary cilia, making it required for leptin signaling in the CNS (194). LepR-expressing cilia are necessary for leptin-mediated neuroendocrine adaptations to starvation and for counterregulatory responses during energy deficiency (195). A recent study identifies that GPR45, colocalized with MC4R in PVN cilia, promotes the activation of the leptin-melanocortin signaling pathway by recruiting Ga_s_ (196). In contrast, ciliary GPR75 regulates feeding behavior and body weight independent of leptin signaling (197).

## Conclusion and future

Leptin plays a pivotal role in energy homeostasis, yet leptin resistance remains a major challenge in obesity treatment. While modifying lifestyle, such as reducing caloric intake, increasing dietary fiber and nutrients, and increasing physical exercise, contributes to body weight management, these behavioral measures are not sufficient to treat obesity or improve leptin resistance. The intricate interactions between leptin and GPCR signaling pathways offer promising therapeutic opportunities for restoring leptin sensitivity and improving metabolic health. Combining leptin with GPCR ligands, particularly gut peptides and their analogs, has shown potential in overcoming leptin resistance and enhancing metabolic outcomes. Despite these advances, several key questions remain. The precise molecular mechanisms underlying GPCR-mediated modulation of leptin signaling still require further investigation, particularly regarding specific neuronal subpopulations and peripheral metabolic tissues. Additionally, understanding the long-term effects of GPCR-targeted combinatorial therapies on weight regulation, metabolic health, and potential adverse effects will be crucial for clinical translation. Furthermore, exploring why certain agents are ineffective in some patients and developing tailored therapies for distinct patient subgroups is essential. Future studies should also focus on novel approaches to sustain weight loss and prevent weight rebound, which would help reduce the psychological, physical, and economic burden of lifelong medication. By integrating insights from leptin signaling and GPCR pharmacology, future research for developing more effective and sustainable anti-obesity interventions is growing brighter.

## Conflict of interest

The authors declare that they do not have any conflicts of interest with the content of this article.
